# Supplementary material for: Enhancing the Spermidine Synthase-Based Polyamine Biosynthetic Pathway to Boost Rapid Growth in Marine Diatom Phaeodactylum tricornutum
Source: Biomolecules. 2024 Mar 19;14(3):372. doi: 10.3390/biom14030372 (PMC10968388; doi:10.3390/biom14030372)
Supplement: Supplementary file 1 [file biomolecules-14-00372-s001.zip › 240321_Diatom_SDS_Supplementary_Information.pdf]

Supporting Information

Enhancing the Spermidine Synthase-Based Polyamine Biosynthesis Pathway to Boost Rapid Growth of Marine Diatoms *Phaeodactylum tricornutum*

Hung-Yun Lin [1], Chung-Hsiao Liu [2], Yong-Ting Kang [2], Sin-Wei Lin [2], Hsin-Yun Liu [2], Chun-Ting Lee [2], Yu-Chen Liu [2], Man-Chun Hsu [2], Ya-Yun Chien [2], Shao-Ming Hong [2], Yun-Hsuan Cheng [2], Bing-You Hsieh [2], Han-Jia Lin [1,2]*

[1] Center of Excellence for the Oceans, National Taiwan Ocean University, Keelung 202301, Taiwan
[2] Department of Bioscience and Biotechnology, National Taiwan Ocean University, Keelung 202301, Taiwan
* Correspondence: hanjia@ntou.edu.tw

Table of Contents:

Table S1. HPLC gradient conditions for PA analysis

Table S2. Information on the specific primers required for clone

Table S3. Specific primers required to verify gene expression in this work

Table S4. The pulse regime of multi-pulse electroporation

Table S5. Amino acid sequence similarity analysis of PtSDS1 and PtSDS2

Table S6. 3D structure similarity analysis of the AlphaFold predicted models of PtSDS1 and PtSDS2 against PDB25 database using DALI server.

List of Figures:

Figure S1. The standard curves for each PA employed in this work

Figure S2. Quantitative PCR standard curves of *RPS*, *PtSDS1*, *PtSDS2*, and *PtCycB1*

Figure S3. SDS-PAGE analysis of purified recombinant proteins

Figure S4. Protein-protein interactions analysis between PtSDS1 and PtSDS2

Table S1. HPLC gradient conditions for PA analysis

ACN	65%	75%	75%	85%	85%	100%	100%	65%	65%
**min**	2.5	3	15	16	22	37	47	47.1	50

Table S2. Information on the specific primers required for clone

Plasmid	Vector	Insert	Sequence (5' to 3")	Mer (bp)	T_m (℃)	Amplicon size (bp)
**pGEX-PtSDS1-His**	pGEX-2T	PtSDS1	gtggatccccgggaattcATGAGCGCTGACGAAGATTCCTC[a]	41	61	897
			gtggtgctcgagcggaaagatggcacGCTGC	31	61	
		His tag	gtgccatctttccgctcgagcaccacCACCACCACCAC	38	74	18
			cagatcgtcagtcagtcacgatGTGGTGGTGGTGGTGGTG	40	72	
**pGEX-PtSDS2-His**	pGEX-2T	PtSDS2	ggatccccgggaattcATGTGGCCGGGTCAAAAATTTTCG	40	61	825
			gtgctcgagctctaatcttgcGGTGACGAAGGGAG	35	62	
		His tag	gcaagattagagctcgagcacCACCACCACCACCAC	36	71	18
			gatcgtcagtcagtcacgatGTGGTGGTGGTGGTGGTG	38	71	
**pGEX-PtSDS1 (Y79F)-His**	pGEX-2T	PtSDS1	gtggatccccgggaattcATGAGCGCTGACGAAGATTCCTC	41	61	249
			ctcttgaaacgcaaattcgtcacG	24	60	
			gtgacgaatttgcgtttcaagagATGATT	29	60	671
			ggtggtggtggtgctcgagCGGAAAGATGGCACGCTGC	38	61	
**pGEX-PtSDS2 (F79Y)-His**	pGEX-2T	PtSDS2	ggatccccgggaattcATGTGGCCGGGTCAAAAATTTTCG	40	61	178
			catgatatgcaaattcATCACGTTCGG	27	60	
			gaatttgcatatcatgAAATGATGGTTCA	29	58	663
			gtggtggtggtgctcgagCTCTAATCTTGCGGTGACGAAGG	41	59	
**pET-Myc-PtSDS1-His**	pET-21a	Myc tag	ggtcgcggatccgaattcGAACAAAAACTCATCTCAGAAGAGGATCTG	48	70	30
			cagcgctcatcatatgcagATCCTCTTCTGAGATGAGTTTTTGTTC	46	68	
		PtSDS1	ctgcatatgatgagcgctgACGAAGATTCCTC	32	61	897
			gtggtggtggtgctcgagCGGAAAGATGGCACGCTGC	37	61	
**pNR-PtSDS1-EGFP**	pNR-EGFP	PtSDS1	cttgtgcgaacggaattcATGAGCGCTGACGAAGATTCCT	40	61	897
			ccttgctcaccataccggtCGGAAAGATGGCACGCTGC	38	61	
**pNR-PtSDS2-EGFP**	pNR-EGFP	PtSDS2	cttgtgcgaacggaattcATGTGGCCGGGTCAAAAATTTTCG	42	61	825
			cttgctcaccataccggtCTCTAATCTTGCGGTGACGAAGG	41	60	
**pNR-PtSAMDC-EGFP**	pNR-EGFP	PtSAMDC	cacttgtgcgaacggaattcATGTCTCCGCTGCCACCGGATTG	43	66	1458
			ctcgcccttgctcaccataccggtCGAAACCGACATGCCCCGCAAC	46	66	
**pNR-PtSAMDC-His**	pNR-EGFP	PtSDS1	cttgtgcgaacggaattcATGTCTCCGCTGCCACCGGA	38	65	1458
			gtgactagtcgaaaccgacATGCCCCGCAA	30	65	
		His tag	gtcggtttcgactagtcacCACCACCACCACCAC	34	70	18
			gcacgcttctgaagcttGTGGTGGTGGTGGTGGTG	35	72	

[a] Lowercase denotes the overlapping region necessary for Gibson assembly.

Table S3. Specific primers used to verify gene expression in this work

Amplified gene	Primer sequence	Mer (bp)	T_m (℃)	Amplicon size (bp)
**RPS**	5′-ATAACTGCACCCACTTCCCA-3′	20	60	
	5′-TGGACCATCTTCACTACGGG-3′	20	62	
**PtCycB1**	5′-GCATCCACGTGTTGGCTCA-3′	19	62	
	5′-CTCCAGCCTACTCATTGGGATCA-3′	23	64	
**PtSDS1**	5′-TAACCTGCTCGAAGACAGAC-3′	20	60	
	5′-AGAGCCATCCGGTGATGTTA-3′	20	60	
**PtSDS2**	5′-GCGACTTCAACAGCAACTC-3′	19	60	
	5′-GGTCCAGGGTTTCTTACTCC-3′	20	62	
**EGFP**	5′-ATAACTGCACCCACTTCCCA-3′	20	60	
	5′-TGGACCATCTTCACTACGGG-3′	20	62	
**PtSAMDC-His**	5′-GCATCCACGTGTTGGCTCA-3′	19	62	
	5′-CTCCAGCCTACTCATTGGGATCA-3′	23	64	

Table S4. The pulse regime of multi-pulse electroporation

	Initial voltage	Pulse time	Pulse interval	Voltage decay rate	Number of pulses
**Poring pulses**	300 V	50 ms	50 ms	10%	8
**Transferring pulses**	8 V	50 ms	50 ms	40%	5

Table S5. Amino acid sequence similarity analysis of PtSDS1 and PtSDS2.

	**PtSDS1**	**PtSDS2**	**HsSDS**	**AtSDS**	**HsSMS**	**AtSMS**
**PtSDS1**	-	48%	58%	50%	26%	50%
**PtSDS2**	48%	-	47%	46%	31%	44%

Table S6. 3D structure similarity analysis of the AlphaFold predicted models of PtSDS1 and PtSDS2 against PDB25 database using DALI server.

N-terminal domain of PtSDS1 (residue 14-74)							
Rank	PDB[1]	Z-score[2]	RMSD[3]	lali[4]	nres[5]	%id[6]	Description
1	4YUV	11.8	1.0 Å	61	294	56	SPERMIDINE SYNTHASE, PUTATIVE;
2	1UIR	7.6	1.2 Å	53	313	36	POLYAMINE AMINOPROPYLTRANSFERASE;
3	2CMG	7.3	1.7 Å	52	262	17	SPERMIDINE SYNTHASE;
4	3C6K	6.5	2.3 Å	53	348	19	SPERMINE SYNTHASE;
5	6SJ9	3.9	2.6 Å	49	642	8	PROTEASOME ACCESSORY FACTOR B/C (PAFBC);
**N-terminal domain of PtSDS2 (residue 21-102)**							
Rank	PDB	Z-score	RMSD	lali	nres	%id	Description
1	4YUV	7.8	0.7 Å	82	294	49	SPERMIDINE SYNTHASE, PUTATIVE;
2	1UIR	7.0	0.7 Å	82	313	45	POLYAMINE AMINOPROPYLTRANSFERASE;
3	3C6K	5.8	1.7 Å	76	348	36	SPERMINE SYNTHASE;
4	2CMG	5.6	1.4 Å	79	262	20	SPERMIDINE SYNTHASE;
5	7UJ5	4.8	2.8 Å	57	257	9	GLUTAMATE RACEMASE;

[1]Resource database: Dail-PDB25 subset Protein Data Bank.

[2]Z-score: How likely you are to find this hit; Z-score less than 4 is usually meaningless.

[3]RMSD: The root-mean-square deviation of atomic positions of Cα between two protein models; RMSD less than 4 Å is usually meaningless.

[4]lali: Length of alignment.

[5]nres: Residues of whole protein.

[6]%id: Protein identity

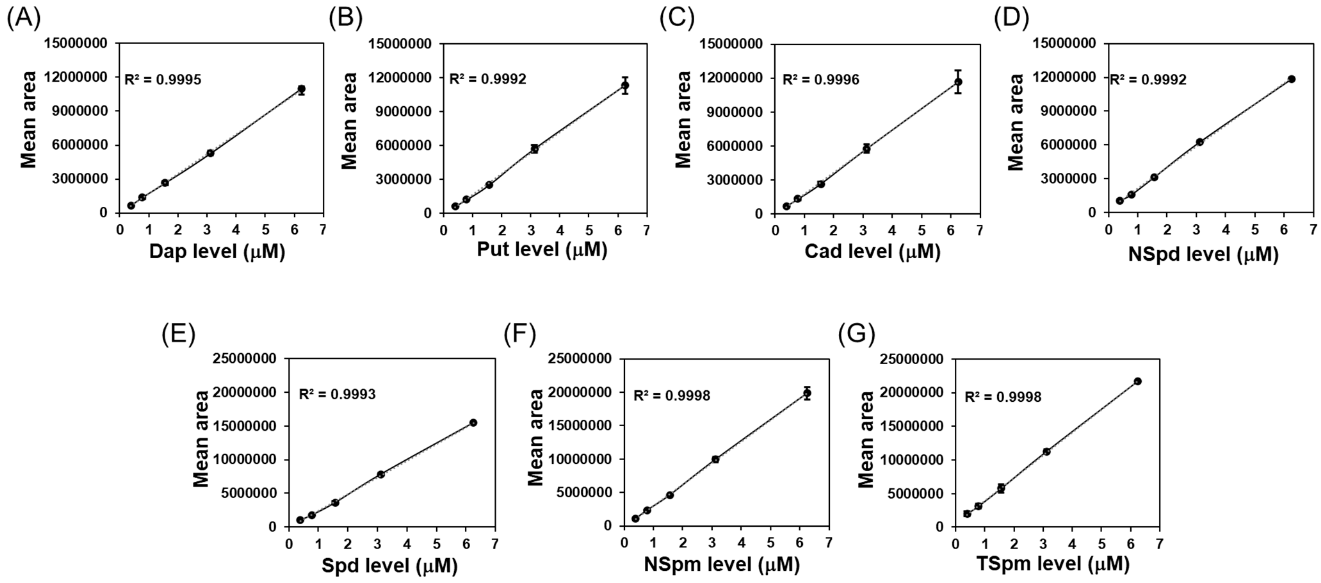

Figure S1. The standard curves for each PA employed in this work. Error bars depicted the standard deviation from the mean of 3 independent experiments.

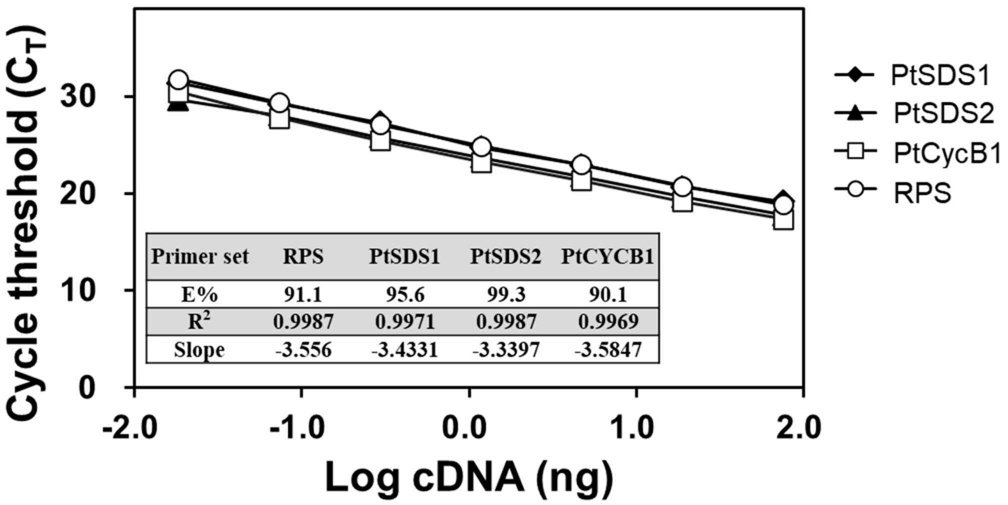

Primer set	RPS	PtSDS1	PtSDS2	PtCYCB1
E%	91.1	95.6	99.3	90.1
R^2	0.9987	0.9971	0.9987	0.9969
Slope	-3.556	-3.4331	-3.3397	-3.5847

Figure S2. Quantitative PCR standard curves of RPS, PtSDS1, PtSDS2, and PtCycB1. The standard curves were constructed by plotting cycle threshold values against the logarithm of serially diluted cDNA. The inset table displays the amplification efficiency (E%), R-squared value (R2), and slope for each gene.

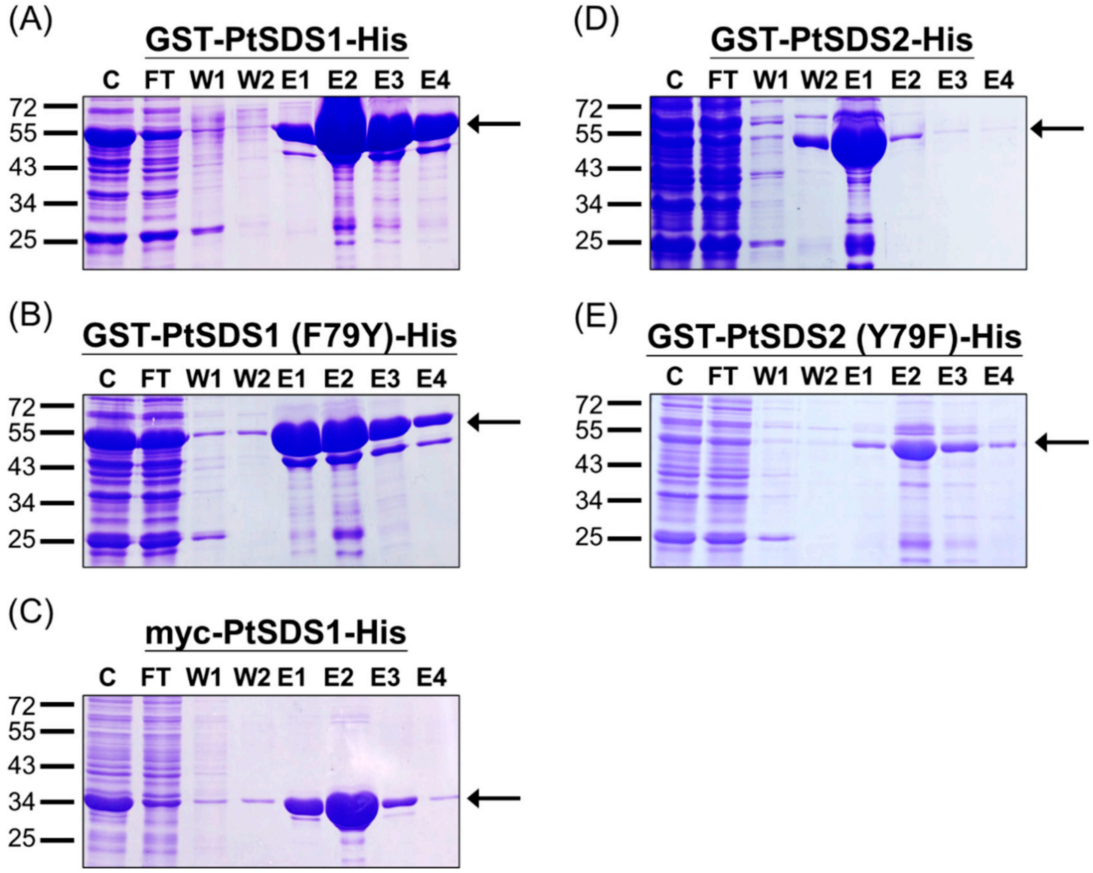

Figure S3. SDS-PAGE analysis of purified recombinant proteins. Recombinant proteins (A) GST-PtSDS1-His, (B) PtSDS1(Y79F), (C) myc-PtSDS1-His, (D) GST-PtSDS2-His, and (E) GST-PtSDS2(F79Y)-His were expressed in *Escherichia coli*, and the target proteins were subsequently purified using Nicke resin. Finally separating recombinant protein through 12% SDS-PAGE, followed by coomassie brilliant blue staining for whole protein visualization. (Meaning of figure's abbr. : Crude protein, C; Flow through, FT; W1/W2, Wash protein 1/2; Elution protein 1-4, E1-E4)

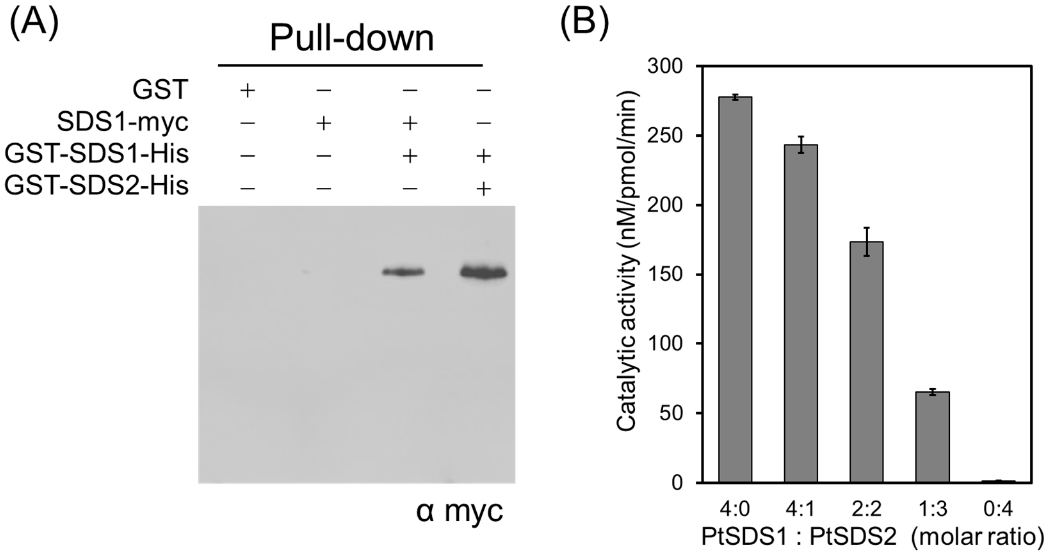

Figure S4. Protein-protein interactions analysis between PtSDS1 and PtSDS2. (A) Analyzing the biochemical properties of recombinant proteins through pull-down assay. Recombinant proteins myc-PtSDS1-His, GST-PtSDS2-His, and GST-PtSDS1-His, each at equimolar concentrations, were mixed and incubated. The resulting protein complexes were then bound to glutathione-Sepharose 4B beads. Following elution with Laemmli sample buffer, the samples were subjected to western blotting analysis. (B) Catalytic activity analysis of the myc-PtSDS1-His and GST-PtSDS2-His recombinant proteins upon mixture. Myc-PtSDS1-His recombinant protein was mixed with GST-PtSDS2-His recombinant protein at various molar ratios (4:0, 3:1, 2:2, 1:3, 0:4), followed by in vitro activity assays in test tubes. Subsequently, the polyamine products were analysed using High-Performance Liquid Chromatography (HPLC).
