# Supplementary material for: Enhancing the Spermidine Synthase-Based Polyamine Biosynthetic Pathway to Boost Rapid Growth in Marine Diatom Phaeodactylum tricornutum
Source: Biomolecules. 2024 Mar 19;14(3):372. doi: 10.3390/biom14030372 (PMC10968388; doi:10.3390/biom14030372)
Supplement: Supplementary file 1 [file biomolecules-14-00372-s001.zip › Additional file S5_Original Images for Blots and Gels.pdf]

Original Images for Blots/Gels

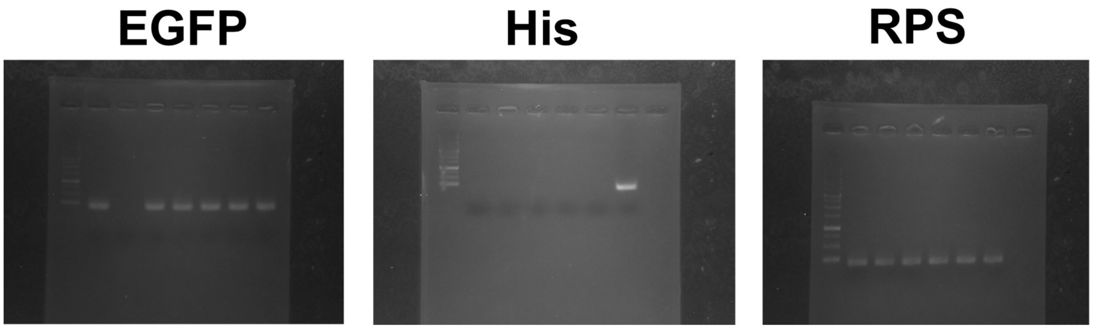

Original agarose gels refer to Figure 5B. In EGFP, lane 2-7 were used for this work.

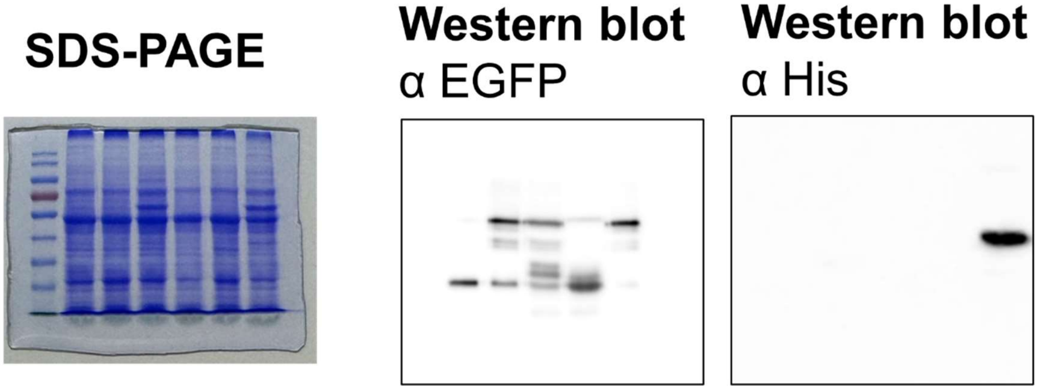

Original western dot photos refer to Figure 5C.

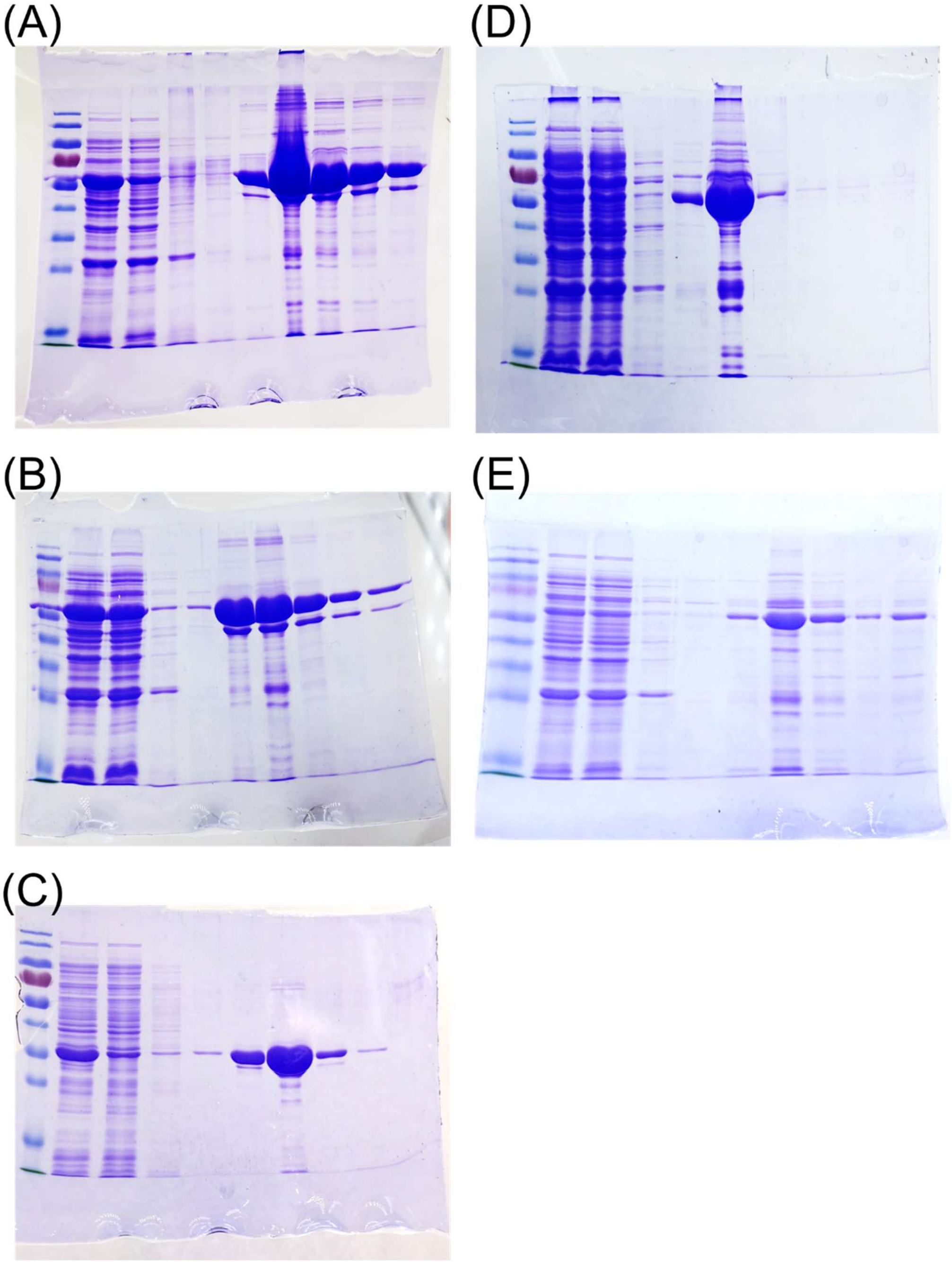

Original SDS-PAGE gels refer to Figure S3 in Supplementary Information.

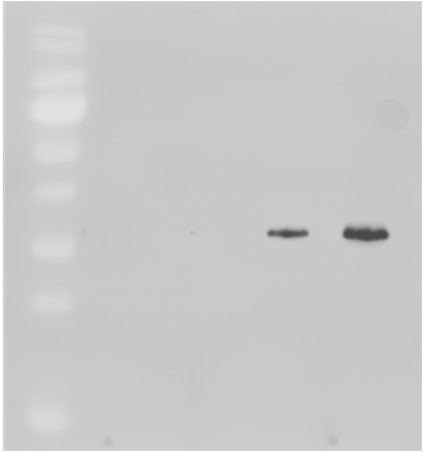

Original western dot photo refers to Figure S4A in Supplementary Information.
